# CurQ+ With Resveratrol Diminish Joint Pain in a Child With Pseudoachondroplasia: A Case Report

**DOI:** 10.7759/cureus.81195

**Published:** 2025-03-25

**Authors:** Jacqueline T Hecht, Ana-Coral Barreda-Bonis, Karen L Posey

**Affiliations:** 1 Pediatrics, McGovern Medical School, UTHealth Houston, Houston, USA; 2 Endocrinologist, Unidad Multidisciplinar de Displasias Esqueléticas, Hospital Universitario La Paz, Madrid, ESP

**Keywords:** curcumin, curq+, joint pain, psach, pseudoachondroplasia, resveratrol, skeletal dysplasia

## Abstract

This case report details the successful use of over-the-counter resveratrol and CurQ+ in a five-year-old with pseudoachondroplasia (PSACH), a severe dwarfing condition caused by mutations in cartilage oligomeric matrix protein (COMP). Disproportionate short stature, abnormal joints, joint deformities, and pain starting in early childhood are characteristic findings. Adult treatments include non-steroidal anti-inflammatory drugs (NSAIDs) and joint replacement to manage joint pain. Childhood pain goes largely untreated given the concern of daily use of NSAIDs in the very young. Recently, resveratrol has been shown to reduce pain and CurQ+ improves growth in a mouse model of PSACH. The use of these over-the-counter supplements has been adopted by some families with PSACH children. One such case in this report has a daily intake of resveratrol and CurQ+ at 125 mg of trans-resveratrol and 0.66 g, respectively, in the 16.6 kg child. This resulted in a dosage of 7.6 mg/kg of resveratrol and 40 mg/kg of CurQ+ daily. To date, no side effects from CurQ+ and resveratrol were reported in the child. Pain made walking to school very difficult (600 m) prior to the use of supplements and now the child walks to school without assistance or complaint.

## Introduction

Pseudoachondroplasia (PSACH) is a well-characterized dwarfing condition marked by disproportionate short stature, joint deformities, and premature joint degeneration, causing lifelong pain [1,2]. A defining characteristic of PSACH is its marked disproportionate short stature, with an adult mean height of 116 cm for females and 120 cm for males [3,4]. Other features include a waddling gait, attractive angular face, extreme joint laxity, and shortened fingers and toes [3]. Although short stature is the most obvious feature, early and painful joint degeneration is the most significant problem for individuals. PSACH prevalence is estimated to be between 1/50,000 and 1/100,000.

Parents of children under five with PSACH report their children rest more, engage in less intense activity, tire easily, and frequently complain of leg pain (personal communication). Pain management in young children is minimal due to the rarity of debilitating joint pain in children and concerns about medication use in this age group. PSACH pain limits mobility and leads to disability and the use of ambulatory aids, and, in short, severely diminishes the quality of life [2].

Chronic pain is frequent, with 81% of 64 PSACH adult participants reporting chronic [2], particularly in the knees, hips, back, shoulders, and legs [2]. This pain adversely affects physical activity, relationships, mood, enjoyment, concentration, sleep, and appetite and is described as miserable, aching, nagging, sharp, throbbing, and exhausting [2].

To manage pain, PSACH adults often require joint replacement surgery before the age of 30 [2,3]. Typically, hips are replaced first, followed by knees and sometimes shoulders [3]. Since the lifespan of adults exceeds that of prosthetic joints, repeated replacements are required. NSAIDs, most commonly meloxicam, provide inadequate pain control, and not all joints can be replaced, leading to substantial untreated pain. Lower limb deformities, such as bowleg, windswept, and knock knee, exacerbate early joint degeneration and require surgical intervention [3]. Collectively, these factors lead to multiple surgeries throughout the life of PSACH individuals. There is a desperate need for non-surgical pain management approaches to improve the quality of life for individuals with PSACH [5].

PSACH is caused by mutations in the cartilage oligomeric matrix protein (COMP) gene [6]. The mutant COMP protein does not fold properly, causing intracellular accumulation that is toxic to the cells that make cartilage called chondrocytes. We have demonstrated in a mouse model of PSACH (MT-COMP mouse) that this intracellular accumulation of misfolded protein includes ER stress, inflammation, and oxidative stress processes that form a self-perpetuating pathological loop [7] suppressing autophagy, leading to senescent articular chondrocytes and joint degeneration characterized by synovitis, inflammation, and subchondral bone remodeling [7-9], all of which have been linked to joint pain in OA. The MT-COMP mouse recapitulates the clinical and chondrocyte pathologies associated with PSACH [10]. In the MT-COMP model, growth is compromised, mice appear smaller than controls by one week of age, and early measurable joint degeneration occurs by 20 weeks of age compared to one year in the background control strain [7]. Preclinical studies in the MT-COMP mouse suggest natural compounds, specifically CurQ+, an easily absorbed formulation of curcumin and liquid resveratrol, may reduce joint pain in PSACH [11,12].

The MT-COMP mice show evidence of pain starting at eight weeks of age. These three assays: 1) gait alterations, 2) reduced voluntary and treadmill running, and 3) reduced grooming (indicative of painful allodynia) demonstrated indirect behavioral evidence of pain in MT-COMP [8,12]. Gait alterations may indicate an attempt to reduce mechanical stress on painful limbs during ambulation [13]. Consistent with painful running, MT-COMP mice showed less voluntary running and 50% less forced treadmill running than controls, likely due to pain with locomotion [7]. Grooming effectiveness, measured by the amount of dye remaining on the back of the neck after a four-hour grooming period [13], showed that at four weeks of age, MT-COMP mice were groomed as effectively as control mice. However, by eight weeks, their grooming effectiveness was significantly reduced [12].

Preclinical studies in the MT-COMP mouse suggest natural compounds, specifically CurQ+, an easily absorbed formulation of curcumin and liquid resveratrol, may reduce joint pain in the MT-COMP mouse model of PSACH [11,12]. Resveratrol is a natural compound found in many foods, including grapes. Curcumin is a compound in turmeric, made from the root of Curcuma longa and used as a cooking ingredient. CurQ+ is a specialized formulation of curcumin that is highly absorbable. Typical doses of resveratrol, 0.25-0.5 g (1 g high), and curcumin, 0.5-2 g (4-8 g high) do not usually cause negative side effects. However, the most common side effect of high doses is gastrointestinal disturbances.

This report presents parental and physician observations from a child with PSACH who was administered over-the-counter liquid resveratrol (Nature’s Answer) and CurQ+ under the supervision of a physician, Ana Coral Barreda, MD. This report offers hope that a non-surgical approach to alleviating PSACH pain can be achieved, potentially improving the quality of life for PSACH individuals.

## Case presentation

Patient history

**Figure 1:**
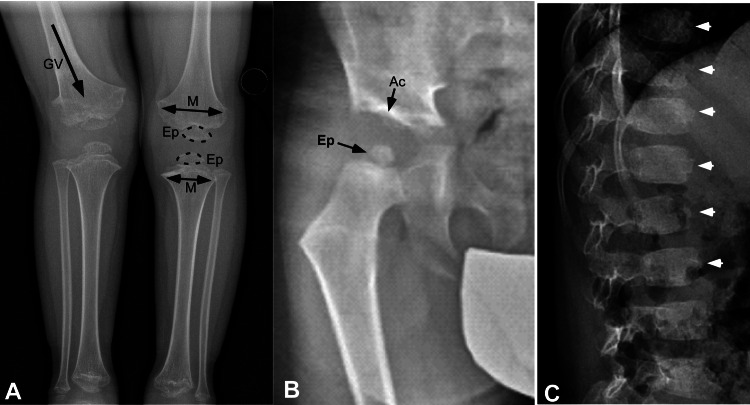
Radiographic findings are consistent with pseudoachondroplasia. (A) Knee image shows small irregular epiphysis (Ep), widened metaphysis (M), and genu valgum (GV). (B) Hips have underdeveloped acetabulum (Ac) and capital femoral epiphyses. (C) Vertebrae show platyspondyly with anterior beaking (marked by white arrowheads).

A five-year-old male was referred to Ana-Coral Barreda-Bonis, MD, for a clinical consult with a presumptive diagnosis of skeletal dysplasia with rhizomelic shortening. At five years, weight was 16.5 kg (-0.96 SD), height 99.7 cm (-2.53 SD), head circumference 54 cm (1.99 SD) [14], Arm-spam to height ratio 0.88. The mother was G2P1 and the pregnancy was conceived by in vitro fertilization. The child was born at 42 weeks after an uncomplicated gestation and vaginal delivery to no-consanguineous parents. Birth weight was 4082 grams (1.07 SD = standard deviation) and a length of 53 cm (0.84 SD) [14]. Parental heights were average. Parents noted failure to thrive from at two years, a right leg limp developed and by 3.5 years a generalized gait disturbance was noted. Right genu valgum (knock-knees) of the right leg developed, and the family consulted an orthopaedist. Radiographs revealed a skeletal dysplasia with small irregular epiphysis of proximal and distal femora, proximal tibia, metaphyseal flaring, and irregular margins with right genu valgum (Figure 1A). Additionally, widened triradiated cartilage, small capital femoral epiphyses, and poorly formed acetabulum were present. There were irregular vertebral bodies with anterior beaking of vertebral bodies (Figures 1B, 1C). Radiologic findings indicate a diagnosis of PSACH. Genetic testing revealed a heterozygous mutation in exon 10 of the COMP gene, which results in a Gly345Arg mutation that has previously been described [15]. Nearby mutations in COMP, Cys348Arg, and Asp342Tyr, are designated as PSACH and severe multiple epiphyseal dysplasia (EDM1), respectively [16,17].

Resveratrol and CurQ+ treatment

Parents chose to administer resveratrol and curcumin daily to the five-year-old child for nine months. CurQ+ gel capsules were allowed to soften in milk and are punctured releasing contents into the milk. Liquid resveratrol (approximately 5 mL containing 125 mg of trans-resveratrol) and cocoa are mixed into the milk served with breakfast. No side effects were noted.

Parental observations

Three months prior to taking resveratrol and CurQ+ and undergoing genu valgus correction surgery at 5.5 years of age, the child appeared to tire easily and was less agile. Before supplementation, the 600-meter walk to school could not be completed, and the parents carried the child. Post-supplementation, the child walks to school without complaint. Jumping and running are now more frequent than before the surgery and supplementation. Complaints about knee pain are less frequent, although knee pain is still present. There are more frequent complaints of pain in the soles of the feet.

Physician observations 

Parents report an improvement in the child’s activity, but the child’s pain has not been measured with a pain scale. Height is 50% for a 2.5-year-old at five years of age. Growth velocity (5 cm per year) and height (-2.7 SD) are maintained but have not worsened.

Resveratrol and CurQ+ information

Nature’s Answer liquid resveratrol containing proprietary resveratrol blend red grape juice concentrate, trans-resveratrol (polygonum cuspidatum 99% root extract), muscadine grape juice extract, muscadine grape pomace extract, red grape pomace extract of 1.3 g (yielding 125 mg of trans-Resveratrol) and proprietary ORAC blend pomegranate juice concentrate, red raspberry juice concentrate, purple corn cob extract 0.7 g. CurQ+ capsules each contain 0.33 g of a propriety blend of the NEM brand of natural eggshell membrane and water-soluble curcumin (curcumin longa) root extract.

## Discussion

This study presents an overview of the clinical and preclinical findings related to PSACH highlighting the potential benefits of natural compounds such as CurQ+ and resveratrol in managing joint pain in this skeletal dysplasia. The observations from both the MT-COMP mouse model [11,12] and the clinical case study of this five-year-old child provide promising evidence for the efficacy of these compounds in reducing pain and improving mobility. We have shown that resveratrol prevents painful joint degeneration in the MT-COMP mouse when administered soon after birth [12]. Other studies have demonstrated that curcumin slows or lessens joint degeneration [18,19], which may diminish inflammation and joint pain. The observations presented here suggest that CurQ+ and resveratrol may reduce leg pain in some PSACH children.

The MT-COMP mouse model of PSACH provides insights into how resveratrol and CurQ+ may alleviate pain. Primarily, both resveratrol and CurQ+ reduce inflammatory markers. Given the link between inflammation and pain, this anti-inflammatory effect likely accounts for most of the short-term pain in the MT-COMP mouse model. Additionally, both compounds mitigate mutant-COMP chondrocyte pathology through their anti-inflammatory and antioxidative properties, as well as by stimulating autophagy to relieve ER stress. This combination of effects may reduce painful synovitis, subchondral bone remodeling, and the establishment of chronic inflammatory processes, thereby diminishing pain over the long term.

It's important to distinguish between anti-inflammatories like ibuprofen and natural compounds that alleviate multiple stresses associated with mutant-COMP pathology. While ibuprofen may reduce pain, it does not address other chondrocyte stresses such as ER stress, oxidative stress, and the inhibition of autophagy. These stresses are crucial to the self-sustaining stress loop initiated by the retention of mutant-COMP, which leads to prolonged ER stress and, ultimately, joint degeneration. This concept has been demonstrated in MT-COMP/CHOP^-/-^ mice, which are deficient in the key ER stress protein CHOP. Moreover, NSAIDs, such as ibuprofen, have more frequent reports of gastrointestinal upset compared to curcumin and resveratrol.

Another essential point is that not all supplement preparations contain the amount of compound listed on the label due to the absence of FDA regulation. Even when comparing the same dose, different supplement preparations are not taken up equally by the body. We have shown that Nature's Answer liquid resveratrol contains the approximate concentration reported on the label and is absorbed by MT-COMP mice. CurQ+ is a specialized formulation of curcumin in coconut oil that dramatically increases uptake. The differences in preparation and absorption of supplements greatly complicate comparisons of studies in the scientific literature. 

Future studies of resveratrol and CurQ+ in the context of PSACH pain should include a systematic clinical study that incorporates comprehensive quality of life and pain assessments to quantify the impact of these interventions on the daily lives of individuals with PSACH, including physical, emotional, and social well-being. Testing the potential synergistic effects of combining CurQ+ and resveratrol with other pharmacological or non-pharmacological interventions could enhance pain management and improve overall outcomes for individuals with PSACH.

Several study limitations impact applicability. The most substantial limitation is that limb alignment surgery occurred three months before the administration of resveratrol and CurQ+, and this surgery would be expected to reduce joint pain. This is a single case report; the findings need to be replicated in a large cohort study. PSACH is a rare condition, thus limiting the number of individuals available at all ages to study safety and efficacy. This is important as resveratrol and CurQ+ supplementation will likely work best in young children who have not lost most growth plates and articular chondrocytes to the underlying pathologic process. Moreover, both CurQ+ and resveratrol are mild blood thinners and should not be used in combination with NSAIDs, which are taken regularly by PSACH adults for pain control. This precludes the participation of most adults in a clinical trial. Nevertheless, long-term studies are required to assess the sustained efficacy and potential long-term side effects of these compounds, which provide hope that a safe, effective, non-surgical approach may be developed to address pain in children with PSACH.

## Conclusions

This is a single case report; the findings need to be replicated in a large cohort study. PSACH is a rare condition, thus, limiting the number of individuals available at all ages to study safety and efficacy. This is important as resveratrol and CurQ+ supplementation will likely work best in young children who have not lost most growth plate and articular chondrocytes to the underlying pathologic process. Moreover, both CurQ+ and resveratrol are mild blood thinners and should not be used in combination with NSAIDs, which are taken regularly by PSACH adults for pain control. This precludes the participation of most adults in a clinical trial. Nevertheless, long-term studies are required to assess the sustained efficacy and potential long-term side effects of these compounds, which provide hope that a safe, effective, non-surgical approach may be developed to address pain in children with PSACH.
